# Unterstützung digitaler Bildungsprozesse durch interaktive gamifizierte Lernvideos – Wie innovative Lernvideos Motivation und Lernerfolg steigern können

**DOI:** 10.1365/s40702-021-00798-w

**Published:** 2021-10-11

**Authors:** Tim Weinert, Dennis Benner, Ernestine Dickhaut, Andreas Janson, Sofia Schöbel, Jan Marco Leimeister

**Affiliations:** 1 Universität Kassel, Kassel, Deutschland; 2 Universität St. Gallen, St. Gallen, Schweiz; 3 Universität Osnabrück, Osnabrück, Deutschland

**Keywords:** Interaktivität, Gamification, Lernvideos, Entwurfsmuster, Interactivity, Gamification, Learning videos, Design pattern

## Abstract

Digitales Lernen ist ein zentraler Bestandteil der heutigen Bildungslandschaft geworden. Insbesondere Lernvideos haben in den letzten Jahren deutlich an Popularität gewonnen und Plattformen wie YouTube sind heute ein fester Bestandteil von Lernprozessen. Bildungseinrichtungen haben diese Entwicklung erkannt und Lernvideos in ihre Lernsysteme integriert. Allerdings sind die heutigen Lernvideos oft nicht sehr ansprechend, weil es an Gestaltungswissen mangelt, wie Interaktionen und gamifizierte Elemente die Motivation und den Lernerfolg steigern können. Dieser Mangel an Gestaltungswissen führt zu minderwertigen Lernvideos, die den Lernprozess der Lernenden meist nicht in dem Maße unterstützen, wie sie es könnten. Um effektive Lernvideos zu entwickeln, welche die Lernenden adäquat unterstützen, ist handfestes und praktisches Gestaltungswissen für die Lehrenden notwendig. Vor diesem Hintergrund entwickeln wir Entwurfsmuster für interaktive gamifizierte Lernvideos, um Praktiker*innen bei der Erstellung effektiver Lernvideos anzuleiten, die die Motivation und das Engagement von Lernenden zu fördern, welche sie aktiv in ihrem Lernprozess unterstützen zu können. Wir setzen damit zusätzlich die Grundlage für zukünftige Forschung zur Gestaltung effektiver Lernvideos. Dabei folgen wir einem gestaltungsorientierten Forschungsansatz, um die Entwicklung unserer Entwurfsmuster zu systematisieren.

**Zusatzmaterial online:**

Zusätzliche Informationen sind in der Online-Version dieses Artikels (10.1365/s40702-021-00798-w) enthalten.

## Abstract (en)

Digital learning has become a core component of today’s educational landscape. Especially modern learning videos have significantly gained in popularity during the last years. Today, platforms like YouTube are an integral part of the learning experience. Educational institutions have recognized this development and incorporated their learning videos into their environment. However, today’s learning videos are often not very appealing because of the lack of design knowledge. This lack of design knowledge results in subpar learning videos that do not support the learning process of learners very well. To develop effective learning videos that adequately support learners, tangible design knowledge for content creators is necessary. Therefore, we develop design patterns for interactive gamified learning videos to guide practitioners in creating effective learning videos that foster motivation and engagement leading to improved learners’ performance, as well as to provide the foundation for future research on the design of effective learning videos. We follow a design science research approach to ensure rigor and relevance of our design pattern.

## Einleitung

Die Popularität des digitalen Lernens wächst exponentiell und insbesondere die Nutzung von Lernvideos nimmt rasant zu. Schon vor der Covid-19-Krise nutzten rund 73 % aller 14- bis 29-jährigen Lernenden Videoplattformen zur Wiederholung von Lerninhalten (Rat für kulturelle Bildung 2019). Speziell der Einsatz von Lernvideos zur Ergänzung bestehender Lernkonzepte kann zu einer Verbesserung des Lernerfolgs führen, wenn sie gut konzipiert und sinnvoll in das bestehende Lehr-Lern-System und deren Lernprozesse integriert sind (Liao et al. 2019; Janson et al. 2020). Obwohl Lernvideos das Potenzial haben selbstgesteuerte Lernprozesse substanziell zu unterstützen, gibt es auch zunehmend Kritik am Einsatz von Lernvideos, da die Nutzung oftmals mit wenig Interaktionen einhergehen. Darüber hinaus wirft der zunehmende Einsatz von Lernvideos die Frage auf, wie den Lehrenden die notwendigen Fähigkeiten vermittelt werden können, um qualitativ hochwertige Lernvideos zu erstellen (Campbell et al. 2020).

Eine Möglichkeit, Lernvideos effektiver zu gestalten, besteht darin interaktive und spielerische Elemente direkt in Lernvideos einzubauen. Interaktive Elemente, wie z. B. ein einfaches Quiz oder Leitfragen, bieten das Potenzial, durch den Einsatz von Funktionen bei der Verwendung von Lernvideos eine bessere selbstgesteuerte Lernerfahrung zu ermöglichen (Brame 2016). Interaktivität verstehen wir in diesem Zusammenhang als Wechselbeziehung zwischen Lernenden und dem Lernmaterial bei der Nutzung der Lernvideos. Die Integration dieser Elemente bietet eine Verbesserung des Engagements und des Lernerfolgs. Dies geschieht zum einen durch die Fokussierung der Aufmerksamkeit der Lernenden auf bestimmte Schlüsselinformationen, welche für den weiteren Lernprozess wichtig sind, zum anderen, um die gezeigten Informationen zu segmentieren, sodass einer Überforderung der Lernenden vorgebeugt wird. Zusätzlich hierzu konnte bereits festgestellt werden, dass diese Interaktionsmöglichkeiten innerhalb von Videos einen positiven Effekt auf die Motivation der Lernenden haben können (Liao et al. 2019). Um die Nutzung der Interaktionselemente weiter zu fördern, können zusätzliche Spielelemente innerhalb der Lernvideos eingesetzt werden, welche besser unter dem Begriff Gamification bekannt sind. Gamification-Elemente können nicht nur die Motivation von Lernenden unterstützen, sondern auch zu besseren Lernergebnissen führen (Ferianda et al. 2018). Während der Einsatz von interaktiven Videos zu Lernzwecken ein zunehmender Trend ist, gibt es unseres Wissens weder in der Forschung noch in der Praxis konkrete Erkenntnisse darüber, wie interaktive Lernvideos gestaltet sein müssen, um ihre Fähigkeiten voll auszuschöpfen. Daher nehmen wir einen Ansatz aus der Systementwicklung, sogenannte Entwurfsmuster, um bewährte Gestaltungslösungen zu kodifizieren. Entwurfsmuster bieten die Möglichkeit, Wissen einfach und nachvollziehbar für Forscher*innen und Praktiker*innen zugänglich zu machen. Vor diesem Hintergrund ist es unser langfristiges Ziel, das Gestaltungswissen über interaktive gamifizierte Lernvideos (IGLV) zu erweitern, um die Lernergebnisse der Lernenden zu verbessern. Daraus folgend möchten wir mit diesem Artikel folgende Forschungsfrage beantworten:

**Wie können interaktive und spielerische Elemente in Lernvideos den Lernerfolg von Lernenden zu verbessern und welchen Beitrag leisten hierfür Entwurfsmuster?**

Um unsere Forschungsfrage zu beantworten, folgen wir einem gestaltungsorientierten (englisch: Design Science Research (DSR)) Ansatz von Peffers et al. (2007), um die Entwurfsmuster systematisch zu entwickeln. Diese Muster sollen dann ihren Beitrag zur Unterstützung von unerfahrenen Lehrenden bei der Entwicklung von hochwertigen IGLV leisten. Der nachfolgende Beitrag ist wie folgt aufgebaut: Zu Beginn werden wir die wichtigsten Grundlagen von IGLV und der Musterentwicklung darlegen. Anschließend werden wir die zugrundeliegende Methodik zur Entwicklung der Entwurfsmuster für IGLV erläutern. Darauf aufbauend werden wir die entwickelten Muster vorstellen und damit Richtlinien für gute Lernvideos näher erläutern. Zum Ende werden wir unsere Ergebnisse diskutieren und auf die Limitationen unserer Arbeit eingehen.

## Theoretischer Hintergrund

### Interaktive Lernvideos

In verschiedenen Disziplinen wie Pädagogik, Psychologie und Soziologie bezieht sich der Begriff „Interaktion“ auf die Wechselbeziehung zwischen Menschen und ihren kommunikativen Aktivitäten mit der Umwelt (Heath und Bryant 2012). Moore (1989) unterscheidet zwischen drei Arten von Interaktionen: Lerner-Inhalt-Interaktion, Lerner-Lerner-Interaktion und Lerner-Lehrer-Interaktion. Wir übernehmen diese drei Arten von Interaktion für unsere Arbeit und definieren Interaktion als Lernaktivitäten, die einen Austausch zwischen Lernenden, Lehrenden und Inhalten beinhalten. Moore (1989) beschreibt die Lerner-Lerner-Interaktion als die Interaktion zwischen den Lernenden selbst. Diese Interaktion ermöglicht den direkten und indirekten Austausch zwischen den Lernenden und fördert zugleich ihre Reflexionsfähigkeit. Die Lerner-Lehrer-Interaktion beschreibt die Interaktion zwischen dem Lernenden und dem Lehrenden. Es ist nicht überraschend, dass die Interaktion zwischen Lernenden und Lehrenden in der Regel die Leistung der Lernenden fördert. Die Interaktion bietet den Lernenden die Möglichkeit, den Lern- und Lehrprozess mit ihren eigenen Ideen und Gedanken aktiv zu gestalten. Die Lerner-Inhalt-Interaktion beschreibt die Interaktion zwischen Lernenden und dem Inhalt. Diese Interaktion kann sodann das Verständnis und die Perspektive des Lernenden verändern, da die Interaktionselemente unterschiedliche Ansätze zum Verständnis des Lerninhalts fördern. Zur Aufbereitung von Lernvideos bieten sich unterschiedliche Interaktionselemente an, welche die Arten von Interaktionen positiv beeinflussen können. Eine Übersicht über verfügbare Interaktionselemente, deren Einsatzmöglichkeiten (beschrieben am Beispiel eines Videos zum Business Model Canvas) und zugrundeliegenden Intention kann Tab. 1 entnommen werden.

**Tab. 1:** Darstellung und Einordnung unterschiedlicher Interaktionselemente

Intention	Interaktionselement	Beschreibung	Beispiel eines möglichen Einsatzszenarios
Bereitstellung von zusätzlichen Informationen	Textuelle‑/Visuelle Einblendung	Einblendung von Informationen auf textueller Basis oder über Video/Bilder	Einblendung eines Bildes zur Verdeutlichung eines Elements im Business Model Canvas (BMC)
	Links	Weiterleitung auf andere Webadresse	Verweis auf ein vertiefendes Video auf Youtube
	Audio-Elemente	Einblendung einer Audiotonspur im Video	Einblenden eines 1‑minuten Potcasts über Cash Flow im BMC
	Animationen	Unterstreichung von Aspekten innerhalb von Videos	Fokus auf einen bestimmten Aspekt im Video über Heranzoomen in einen bestimmten Bereich
Förderung des Austausches	Links^a^	Weiterleitung auf andere Webadresse	Weiterleitung zu einer videobegleitenden Chatgruppe
	Umfragen	Befragung der Studierenden nach ihrer Meinung zu einem bestimmten Problem	Übersicht über die Ansichten der Studierenden zum Aufwand der Entwicklung von Geschäftsmodellen
	Foren	Virtueller Platz zum Austausch und Archivierung von Gedanken und Erfahrungen	Austausch zwischen Studierenden zu einem bestimmten Aspekt im Video
Förderung der Selbstorganisation	Querverweise	Verweise auf andere Videos im Kurs	Verweis auf ein anders Video zur Wiederholung des Aufbaus des BMC
	Kreuzungen/Überspringen	Verweise auf bestimmte Thematik innerhalb des Lernvideos, oft in Zusammenspiel mit einer Kontrolle des Wissenstands	Überspringen des Aufbaus des BMC nachdem ein Wissenstest zur Kontrolle des Vorwissens erfolgreich abgeschossen worden ist
	Links^a^	Weiterleitung auf andere Webadresse	Weiterleitung auf ein Organisationsboard, welche den aktuellen Lernfortschritt über die Lektionen darstellt
	Lesezeichen	Erstellen von Lesezeichen innerhalb des Videos zur Hervorhebung bestimmter Bereiche	Hervorhebung eines Fallbeispiels für den Einsatz des BMC
Kontrolle des Wissensstands	Hotspot	Definierter Bereich im Video, welcher auf eine Webseite oder Timecode verweist	Hyperlink zu einer bestimmten Stelle im Video, wenn auf eine Graphik geklickt wird
	Quiz	Einfache Wissensfragen, welche Wahr oder Falsch sein können	Abfrage des Einsatzszenarios für das BMC
	Lückentexte	Kontrolle zum Test des Wortschatzes oder ganzer Wortfolgen	Erschließung des Kontextes in einer Fallstudie und Füllung mittels Fachvokabular
	Drag & Drop	Füllen einer Graphik mit vorgegebenen Elementen	Aufbau eines BMC mit vorgegebenen Elementen erläutern
	Offene Fragen	Offene Fragen zu Beantwortung von komplexen Sachverhalten	Abwägung der Vor- und Nachteile des Einsatzes des BMC
	Multiple Choice	Eine Frage, bei welcher die Teilnehmenden aus mehreren vordefinierten Antwortmöglichkeiten wählen können	Abfrage des Vorgehens bei der Entwicklung seines eigenen Geschäftsmodells

^a^Können mehreren Intentionen zugeordnet werden

Insbesondere können die Interaktionselemente vier unterschiedliche Intentionen verfolgen, welche die Interaktion innerhalb des Videos fördern können. Erstens bieten Interaktionselemente die Möglichkeit, *zusätzliche Informationen bereitzustellen*, um bestimmte Zusammenhänge mit anderen Quellen zu unterstreichen. Zweitens ermöglichen Interaktionselemente die *Förderung der Kommunikation* durch direkte Kanäle wie Foren oder Chatgruppen zwischen den Lernenden und Lehrenden. Drittens können Interaktionselemente die Studierenden dabei unterstützen, ihren Lernprozess über die *Lernvideos hinweg selbst zu organisieren und zu steuern*. Beispielsweise können Studierende durch Lesezeichen in die Lage versetzt werden, bestimmte für sie wichtige Bereiche im Video schnell zu finden. Viertens ermöglichen Interaktionselemente die Wiederholung und *Kontrolle des erlernten Wissens*, um den Wissensstand der Studierenden zu überprüfen und den Studierenden die Möglichkeit zu geben, ihren Lernprozess selbst zu evaluieren.

Interaktionselemente, die synchrone und asynchrone Interaktion fördern, stimulieren „das Interesse an dem, was gelehrt werden soll, um die Lernenden zum Lernen zu motivieren, um das Interesse des Lernenden zu steigern und aufrechtzuerhalten“ (Moore 1989, S. 2). Andererseits erlauben Interaktionselemente den Lernenden, ihre Aufmerksamkeit auf die wesentlichen Teile des Videos zu richten und helfen so, eine Überlastung der kognitiven Kapazitäten der Lernenden zu vermeiden (Sweller 1994). Dies kann den Lehrenden helfen, den Nutzen der Videos zu maximieren. Die Vermeidung von kognitiver Belastung ist im Gestaltungsprozess von interaktiven Lernvideos wichtig und bietet einen Rahmen für die Gestaltung von Lernmaterialien. Aufgrund der begrenzten kognitiven Kapazitäten können die Lernenden nur einem Teil der angebotenen Informationen Aufmerksamkeit schenken.

### Gamifiziertes Lernen im Kontext von interaktiven Lernvideos

**Abb. 1:**
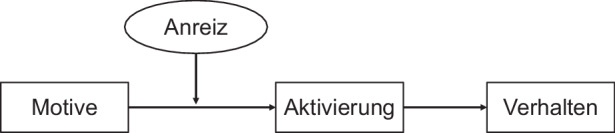
Aktivierung von menschlichen Verhalten. (Angepasst nach Leimeister et al. (2009))

Um die positiven Effekte von interaktiven Lernvideos ausschöpfen zu können, ist es notwendig, Lernende zur Nutzung der Interaktionselemente zu motivieren. Um fehlender Motivation entgegenzuwirken und um die Lernenden zusätzlich zur Nutzung der Interaktionselemente zu animieren, können sich Gamification Elemente als sinnvolle Ergänzung erweisen, um die Motivation der Lernenden zu verbessern. Gamification hat sich bereits in verschiedenen Anwendungen als effektiver Treiber für intrinsische Motivation und damit für die Motivation von Nutzenden erwiesen, um ein erwünschtes Ergebnis z. B. mehr Lernerfolg zu erzielen (Ryan und Deci 2016). Im digitalen Lernen ist Gamification ein bekanntes und etabliertes Konzept, das sich als funktionierendes und wertvolles Mittel zur Förderung der Motivation von Lernenden bewährt hat (Schöbel et al. 2020). Gamification selbst wird als Verwendung von Spiel-Elementen in einem nicht-spielbasierten Kontext verstanden (Deterding et al. 2011). Weiterhin kann Gamification als ein Prozess der spielerischen Gestaltung von Aktivitäten in einem spielfremden Kontext definiert werden (Sailer 2016). Im Kontext von Informationssystemen, unter anderem Lernvideos, können mittels Gamification monotone und langweilige Aufgaben durch spielerische Elemente angenehmer gestaltet werden (Schöbel und Janson 2018). In Bezug auf IGLV heißt dies, den spielfremden Kontext der Lehr‑/Lernprozesse und deren monotone Lernvideos mit motivationalen Komponenten – den Spiel-Elementen – anzureichern, um erwünschte Verhaltensresultate wie das Lernen zu erzielen und damit den Lernerfolg nachhaltig zu fördern (Deterding et al. 2011). Diese Aktivierung von Verhalten kann durch das untenstehende Modell (Abb. 1) nach Leimeister et al. (2009) verdeutlicht werden. Die eingesetzten Spiel-Elemente können dabei als Anreiz für die Aktivierung verstanden werden, um ein bestimmtes Verhalten bei den Studierenden auszulösen.

Um diese Elemente im Rahmen von Lernvideos einsetzen zu können, ist ein tieferer Blick auf die Gestaltung von Lernvideos notwendig. Qualitativ hochwertige IGLV können zu einer Verbesserung der Motivation führen, was sich positiv auf den Lernerfolg auswirken kann. Sowohl interaktives Design als auch Gamification können für sich genommen unser gewünschtes Ergebnis unterstützen. Allerdings haben beide auch ihre Grenzen. Einerseits können interaktive Lernvideos den Lernprozess ansprechend gestalten, aber ohne besondere Features fehlen die Mittel, um Motivation zu erzeugen. Auf der anderen Seite kann Gamification allein die Lernenden nicht ausreichend fesseln, füllt aber die Motivationslücke von interaktiven Lernvideos. Durch das Hinzufügen von gamifizierten Komponenten zu interaktiven Lernvideos, also die Schaffung von IGLV, kann Motivation sichergestellt werden. Folglich kombinieren wir interaktives und gamifiziertes Design, um den Lernerfolg durch Steigerung von Motivation und Engagement zu verbessern und gleichzeitig die kognitive Last zu reduzieren.

### Entwurfsmuster zur Gestaltung von IGLV

Für die Gestaltung von neuartigen Technologien, wie in unserem Fall interaktiven Lernvideos, sollte im Idealfall auf bewährte Lösungen zurückgegriffen werden. Die Systementwicklung macht sich genau dieses Prinzip durch die Nutzung von sogenannten Entwurfsmustern (engl. Design Pattern) zu Nutze. Entwurfsmuster stellen eine Möglichkeit dar, um bewährte Lösungen für wiederkehrende Probleme weiterzugeben. Hierbei bedarf es meist keinen neuen, innovativen Lösungen, sondern einer Kodifizierung von bestehenden und insbesondere bewährten Prinzipien. Je nach Einsatzszenario kann sich die Darstellung von Entwurfsmustern verändern und individuell an den eigenen Zweck angepasst werden. Wichtig ist dabei, dass jedes Entwurfsmuster einen eindeutigen Namen hat, der bereits die Grundidee des jeweiligen Entwurfsmusters beschreibt und den Anwendenden erste Implikationen der Gestaltungslösung gibt. Im Kern jedes Entwurfsmusters sollte das zu lösende Problem beschrieben und mögliche Lösungsansätze aufgezeigt werden, in unserem Fall also wie sich Lernvideos erfolgreich durch Interaktion und Gamification gestalten lassen. Bedeutsam ist dabei, dass der Aufbau jedes einzelnen Entwurfsmusters gleich ist, sodass in möglichst kurzer Zeit die notwendigen Informationen gefunden werden können. Die Kombination mehrerer Entwurfsmuster wird häufig Entwurfsmusterkatalog genannt. Einzelne Entwurfsmuster eines Entwurfsmusterkatalogs können dabei miteinander in Beziehung stehen, weshalb Entwurfsmuster häufig untereinander verlinkt sind.

In diesem Beitrag wollen wir uns das Prinzip der Entwurfsmuster zu Nutze machen, um unser Gestaltungswissen für die Gestaltung interaktiver Lernvideos weiterzugeben. Didaktische Entwurfsmuster sind im Lehr-Lernkontext beispielsweise bereits für die Unterrichtsgestaltung eingesetzt (Bauer 2008) oder aber wie digitale Medien sinnvoll in die Hochschullehre integriert werden können (Vogel und Wippler-Mann 2003). Dazu orientieren wir uns an den Erkenntnissen von Dickhaut et al. (2020) und kodifizieren unser Wissen zur Gestaltung von interaktiven Lernvideos in Entwurfsmustern. Zunächst erarbeiten wir eine Entwurfsmusterschablone, um alle notwendigen Gestaltungsdetails abzudecken, die den Videogestaltenden bestmöglich unterstützen. Durch die Erstellung von Entwurfsmustern wird es ermöglicht das entwickelte Gestaltungswissen in optimal strukturierter und abstrahierter Form weiterzugeben, um es in vielen verschiedenen Anwendungsszenarien nutzbar zu machen. Daher fokussieren wir uns bei den Inhalten nicht auf bestimmte Videotechnologien oder Programme, sondern geben allgemeingültige Empfehlungen für die Videoerstellung.

## Methodische Ansätze in der gestaltungsorientierten Forschung

**Abb. 2:**
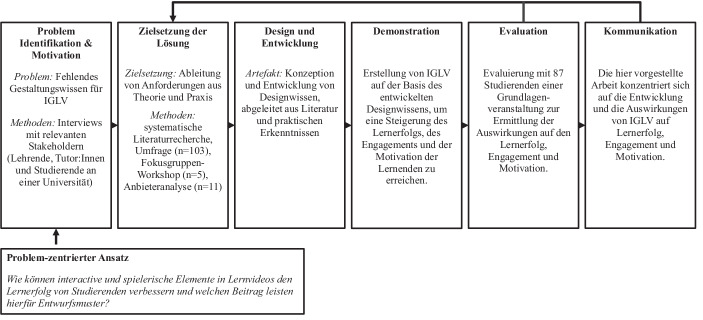
Gestaltungsorientierter Ansatz für die IGLV (in Anlehnung an Peffers et al. (2007)). Im Rahmen dieser Studie werden alle Phasen durchlaufen, jedoch wird nur eine Iteration durchgeführt

Der Forschungsansatz folgt einem problemzentrierten, gestaltungsorientierten Ansatz, wie er von Peffers et al. (2007) eingeführt wurde, um Gestaltungswissen über IGLV in Entwurfsmuster zu kodifizieren (siehe Abb. 2). Wir beginnen unser DSR-Projekt mit einem problemzentrierten Ansatz, da unsere Forschung von den bestehenden Problemen bei der Erstellung von Videoinhalten und der Kommunikation ihres Gestaltungswissens angetrieben wird. Um unseren Entwurfsmusterkatalog zu entwickeln, folgen wir der Entwurfsmustervorlage von Dickhaut et al. (2020). Anschließend werden diese Entwurfsmuster im Rahmen einer Grundlangenveranstaltung an einer Universität evaluiert.

### Erhebung von Anforderungen aus Theorie und Praxis

Um die notwendigen Anforderungen aus Theorie und Praxis für die erste Iteration zu erheben, haben wir eine Anforderungserhebung in mehreren Schritten durchgeführt, um das notwendige Gestaltungswissen für die Konzeption unserer IGLV ableiten zu können. Hierzu wurden zum einen theoretische Anforderungen aus der Literatur sowie praktische Anforderung mittels Umfragen, Fokusgruppenworkshop und Anbieteranalyse abgeleitet.

#### Systematische Literaturanalyse

Im ersten Schritt leiteten wir Anforderungen ab, die sich auf die identifizierten Konzepte der Interaktion (Moore 1989) und Gamification (Deterding et al. 2011; Hamari et al. 2014) beziehen. Dazu haben wir eine Literaturrecherche nach den vorgeschlagenen Methoden von Webster und Watson (2002) sowie Vom Brocke et al. (2015) durchgeführt, um relevante Theorien und Designs zu identifizieren, auf die wir bei der Erstellung unserer Anforderungen zurückgreifen können. Für die Suche nutzten wir die folgenden gängigen Datenbanken bzw. Suchmaschinen: ACM, AISeL, IEEEXplore, JSTOR, SSRN, ScienceDirect, SpringerLink und EBSCOhost. Die Auswahl der Datenbanken spiegelt eine breite Pallette verschiedener Literatur und Forschungsströme wider, darunter eher technisch-informatisch orientierte Vertreter wie ACM oder IEEEXplore und qualitativ herausragende Vertreter inkl. des *Basket of Eight* über die AISeL. Für die Datenbanksuche benutzten wir die Suchwörter „gamification“, „nudging“, „videos“, „learning“, „education“, „training“ und „interactive“. Wir kombinierten die Wörter und nutzten, wo möglich, Wildcards, woraus folgender Suchstring resultierte: (gamif* OR nudg* OR interact*) AND (learn* OR edu* OR train*) AND video*. Gemäß dem Falle, dass eine Datenbank keine Wildcards unterstützt oder andere Einschränkungen nötige waren, haben wir den Suchstring den Fähigkeiten der Datenbank bzw. Suchmaschine anpassen müssen. Bei der primären Datenbanksuche haben wir uns ausschließlich peer-reviewed Quellen aufgenommen, bei der weiteren Vorwärts‑/Rückwärtssuche nach Webster und Watson (2002) fiel diese Einschränkung weg.

In einem ersten Schritt konnten wir 1596 Studien identifizieren. Nach der Analyse des Titels, der Zusammenfassung und der Schlüsselwörter jedes Artikels und der vollständigen Untersuchung potenziell einflussreicher Artikel konnten wir 35 relevante Artikel identifizieren, von denen sich 27 auf Interaktivität und nur 8 auf Gamification von Lernvideos bezogen. Diese ungleiche Verteilung, auf die wir bei unserer Literaturrecherche gestoßen sind, könnte auch eine potenzielle Forschungslücke bezüglich der Anwendung von Gamification bei Lernvideos aufzeigen.

#### Umfrage und Fokusgruppenworkshop

Um unsere Erkenntnisse aus der systematischen Literaturrecherche zu bereichern, haben wir eine Umfrage und eine Fokusgruppe unter Universitätsstudierenden durchgeführt, die bereits interaktive und nicht-interaktive Lernvideos verwendet haben. Im Rahmen der Studie wurden 103 Studierende gebeten, Vorschläge zur Verbesserung der bestehenden Lernvideos für eine Wirtschaftsinformatik-Vorlesung zu machen, woran sich insgesamt 93 Studierende beteiligt haben. So wurde den Studierenden anonym die Möglichkeit gegeben, ihre Meinung zu einem bestehenden (interaktiven) Lernvideo zu äußern und Vorschläge für eine bessere Umsetzung der Interaktionselemente zu machen. Die Lernvideos waren mit verschiedenen Interaktionselementen, wie z. B. einfachen Quizfragen, Sprungpunkten oder integrierten Bildern ausgestattet. Insbesondere wurde von den Studierenden festgestellt, dass Lernkontrollen (z. B. Quizze) gut platziert und verständlich präsentiert werden müssen. Neben offensichtlichen Rückmeldungen hinsichtlich technischer Mängel in den Videos (Audio und Videoqualität) wurde insbesondere die fehlende Struktur und der Aufbau der Interaktionselemente kritisiert. Konkret wurde von mehreren Studierenden angemerkt, dass die Interaktionselemente nicht aufeinander abgestimmt wären und nicht erkennbar auf die Lernziele des Videos bzw. des jeweiligen Moduls abzielen würden.

Basierend auf diesen Erkenntnissen führten wir einen ergänzenden Fokusgruppen-Workshop mit fünf Studierenden (m = 4, w = 1) des Wirtschaftsingenieurwesens und des Maschinenbaus durch. Während des Workshops wurden die Teilnehmenden gebeten, ihre Vorstellungen von einem guten Lernvideo zu entwickeln, die vorhandenen interaktiven Lernvideos zu bewerten und Hinweise zu deren Verbesserung zu geben. Mit Hilfe der Brainstorming-Elemente von Briggs und de Vreede (2009) wurden die aufkommenden Kategorien ermittelt, priorisiert und zusammen mit der Umfrage in die abschließenden Anforderungen eingebettet. Dabei wurden die Anmerkungen der Studierenden in Hinweise hinsichtlich der Verständlichkeit, der Visualisierung, der Länge und der Interaktionsmöglichkeiten in den Videos eingeordnet, um einen besseren Überblick über die verschiedenen Anmerkungen zu erhalten. Während des Workshops wurden die Teilnehmenden gebeten, ihre Vorstellungen von einem guten Lernvideo zu entwickeln, die vorhandenen interaktiven Lernvideos zu bewerten und Hinweise zu deren Verbesserung zu geben. Anschließend wurden die so generierten Ideen und Anmerkungen der Studierenden von den Teilnehmenden innerhalb der Kategorien priorisiert, um die für die Studierenden relevantesten Anmerkungen zu kanalisieren zu können (Briggs und de Vreede 2009).

#### Systematische Anbieter-Analyse

Um zusätzliche Informationen über die Funktionalitäten interaktiver Lernvideos zu gewinnen, haben wir eine systematische Plattformevaluation von existierenden Videoplattformen durchgeführt. Hierzu haben wir mittels der Suchplattformen Google, Bing und Yahoo nach Anbietern gesucht, welche die Integration von interaktiven und gamifizierten Elementen innerhalb von Lernvideos ermöglichen. Tab. 2 zeigt eine Übersicht über die verschiedenen Anbieter.

**Tab. 2:** Übersicht über die identifizierten Anbieter

Nr	Name des Anbieters	Identifizierte Interaktionselemente
1	ThingLink	Textliche/visuelle Elemente, Beschriftungen, Links, Querverweise/Touren zu anderen Videos
2	Wirewax	Textliche/visuelle Elemente, Hotspots
3	Vidzor	Textliche/visuelle Elemente, Hotspots, Kreuzungen, Überspringen, Links
4	Storygami	Videoelemente
5	Metta	Textliche/visuelle Elemente, Audio-Elemente, Quiz, Leaderboards
6	Playposit	Quiz, MC-Fragen, offene Antwortfragen, Umfragen, Lückentexte, Foren
7	Touchcast	Textliche/visuelle Elemente, Dokumente, Links
8	Wootag	Textliche/visuelle Elemente, Hotspots
9	Wideo	Textliche/visuelle Elemente, Audioelemente, Animationen
10	Camtasia	Textliche/visuelle Elemente, Audio-Elemente, Quiz, Umfragen
11	H5p	Textliche/visuelle Elemente, Kreuzungen, Quiz, MC-Fragen, Drag & Drop, Lückentexte

Hier folgten wir den drei von Yin (2003) vorgeschlagenen Prinzipien der Datenerhebung. Um mögliche Anbieter interaktiver Lernvideos zu identifizieren, nutzten wir zwei Suchplattformen (Google und Bing). Wir wählten 11 Anbieter verschiedener Arten von (interaktiven) Elementen für die Videobearbeitung aus, die auf unterschiedliche Arten der Interaktion abzielen. Dies ermöglichte es uns, die Funktionalitäten von interaktiven Lernvideos für eine Vielzahl von Anwendungsbereichen zu untersuchen.

#### Anforderungen an IGLV

Ausgehend von den Erkenntnissen aus der systematischen Literaturrecherche und den praktischen Erhebungen aus der Umfrage, dem Fokusgruppenworkshop sowie der Anbieteranalyse soll im nächsten Schritt eine Anforderungserhebung für die nutzerorientierte Gestaltung von IGLV stattfinden. Eine genauere Herleitung der Forschungslücken und Anforderungen kann auch Weinert et al. (2020) entnommen werden. Eine Zusammenfassung der Anforderungen wird in Tab. 3 dargestellt und anschließend erläutert.

**Tab. 3:** Anforderungen aus Theorie und Praxis an die Gestaltung von IGLV

Anforderung	Erläuterung
Anf. 1: Lernziele	Verwenden Sie die Lernziele, um die Positionen der interaktiven Elemente zu bestimmen, indem Sie interaktive Elemente an den Stellen hinzufügen, an denen die Lernziele im Video angesprochen werden
Anf. 2: Lernkontrollen	Verwenden Sie Lernkontrollen am Ende des Videos, um zu überprüfen, ob der Inhalt verstanden wurde
Anf. 3: Freiwillige Bewertung	Verwenden Sie von Anfang an Elemente der Interaktion, damit die Lernenden freiwillig tiefer in das Thema einsteigen und ihren Lernfortschritt überwachen können
Anf. 4: Förderung des Austausches	Überlegen Sie, wo eine Aufgabe zur Interaktion mit anderen Kursteilnehmenden sinnvoll und notwendig ist und fügen Sie an dieser Stelle Elemente ein, die die Kursteilnehmenden zur Interaktion miteinander einladen
Anf. 5: Einbeziehung motivierender Elemente	Binden Sie Elemente des Spieldesigns ein (z. B. ein Belohnungssystem wie Punkte oder Abzeichen, die gesammelt werden können), um die Lernenden zu motivieren, interaktive Videoelemente zu nutzen und sich an der freiwilligen Interaktion zu beteiligen

Im Rahmen der Analyse wurde schnell deutlich, dass es bei der Nutzung der IGLV oftmals ein Überforderungserleben bei den Studierenden auftritt, wenn zu viele Interaktionselemente gleichzeitig eingesetzt werden. Zudem stellten sich viele Studierende die Frage, welcher Zusammenhang zwischen den Interaktionen und dem jeweilig im Video gezeigten Thema besteht. Gleichzeitig betonen mehrere Studien die Bedeutsamkeit von Lernzielen zur Orientierung von Lernenden innerhalb des Lernprozesses (Brown et al. 2016). Vor diesem Hintergrund sollen für die richtige Platzierung der Interaktionselemente die Lernziele berücksichtigt werden (Anf. 1).

Als Interaktionselemente eignen sich besonders Gliederungselemente wie Kreuzungen, die für die Lernenden wichtige Bereiche im Video markieren. Aufgrund von fehlenden Interaktionen im Lernvideo ist es oft schwierig zu beurteilen, ob die Lernenden die Lerninhalte verstanden hat oder nicht. Solche obligatorischen Lernfortschrittskontrollen, die durch Fragetypen wie Multiple-Choice-Fragen oder Drag-and-Drop-Elemente ermöglicht werden, sollten eher am Ende des Lernvideos eingesetzt werden, damit der Lernfluss nicht unnötig gestört wird (Anf. 2).

Auf der anderen Seite sollten die Lernenden in die Lage versetzt werden selbstständig zu überprüfen, ob die gezeigten Inhalte wirklich verstanden wurden. Diese freiwilligen Lernfortschrittskontrollen können durch verschiedene Interaktionselemente gezielt ermöglicht und während des gesamten Lernvideos angeboten werden, da diese von den Lernenden aktiv angeklickt werden müssen und das Video nicht automatisch stoppen. Diese Checks dienen der Selbsteinschätzung der Lernenden und bieten ihnen somit die Möglichkeit, ihre Lernprozesse kritisch zu reflektieren. Außerdem wird das Abschweifen der Gedanken verhindert, da sich die Lernenden kontinuierlich mit den Inhalten auseinandersetzen müssen (Schacter und Szpunar 2015), ohne ihren Lernprozess aktiv zu unterbrechen (Anf. 3).

Die Reflektion des Lernens erfordert einen Austausch zwischen den Lernenden. Wie Coetzee et al. (2015) festgestellt haben, können bereits kurze Interaktionen zwischen den Lernenden dazu beitragen ihren Lernerfolg zu verbessern. Durch die Verwendung von wiederkehrenden Bildern, die die Lernenden dazu auffordern, sich in einer bestimmten Art und Weise zu verhalten, oder mittels Links zu externen Quellen wie Foren oder Chats, kann der Austausch zwischen den Lernenden gefördert werden. Deshalb sollten Interaktionselemente innerhalb der Lernvideos diesen kurzen Austausch fördern (Anf. 4).

Um die Lernenden nachhaltig zum Lernen mit IGLV zu motivieren und insbesondere die Nutzung der Wissenstests zur Selbstkontrolle zu fördern, werden motivatorische Gestaltungselemente empfohlen (Anf. 5). Aus einem Workshop inkl. Fokusgruppe mit Studierenden ging hervor, dass diese motivatorischen Gestaltungselementen gegenüber sehr zugetan waren und betonten, diese würden sie zur Nutzung der Videos und vor allem der Wissenstests in Form von Quizzen motivieren. Als motivatorische Gestaltungselemente sind Spiel-Design-Elemente besonders gut geeignet. Beispielsweise Abzeichen und Punkte (in diesem Fall Sterne) werden hierbei für abgeschlossene Wissenstests vergeben und können am Ende eines jeden Lernvideos als Übersicht eingesehen werden.

### Entwurfsmuster für die Entwicklung von IGLV

**Abb. 3:**
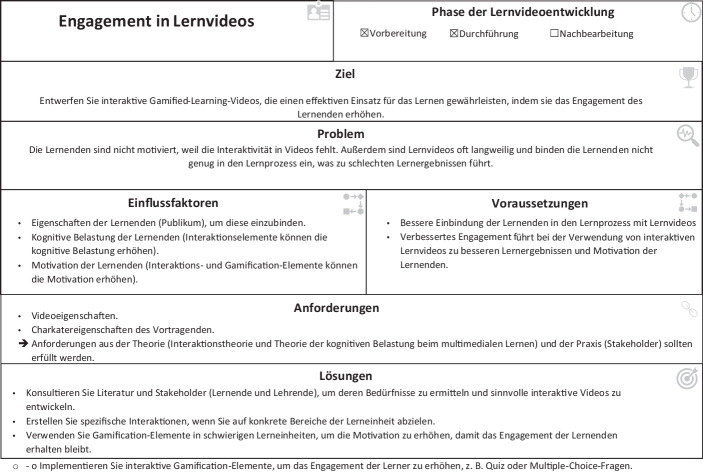
Beispielhaftes Entwurfsmuster für die Verbesserung des Engagements in Lernvideos

Die erhobenen Anforderungen zur Erstellung hochwertiger IGLV und zur Unterstützung von Praktiker*innen stellen die Grundlage für die Erstellung der Entwurfsmuster dar. Die Entwurfsmuster sollen Lehrende bei der Entwicklung ihrer eigenen Lernvideos unterstützen. Gleichzeitig können durch die durchgeführte Anbieter-Recherche die Möglichkeiten und Grenzen des momentanen Standes der Technik abgeschätzt werden, welche die Einsatzmöglichkeiten von interaktiven und spielerisch gestalteten Lernvideos begrenzt. Entwurfsmuster bieten dabei eine etablierte Möglichkeit komplexes Gestaltungwissen einfach und praxisnah übersetzen zu können. Um den grundsätzlichen Aufbau unserer Entwurfsmuster zu erläutern, nutzen wir beispielhaft das Entwurfsmuster „Engagement in Lernvideos“, welches zur Verbesserung des Engagements innerhalb von Lernvideos erstellt wurde (siehe Abb. 3).

Um den eindeutigen Anwendungszweck der IGLV zu beschreiben, erhält jedes Entwurfsmuster einen eindeutigen Namen (z. B. „Engagement in Lernvideos“), der bereits Rückschlüsse auf den Inhalt des Entwurfsmusters zulässt. Das Entwurfsmuster wird unter „Phase der Lernvideoentwicklung“ in Bezug auf seine Anwendung während des Videoentwicklungs- und Lernprozesses klassifiziert. Weiterhin wird es dann durch das Ziel und das Problem charakterisiert, das es zu lösen versucht. In unserem Beispiel ist es das Ziel, das Engagement und die Motivation der Lernenden zu verbessern, indem Gestaltungselemente innerhalb der Lernvideos so angepasst werden, dass diese positiv auf die Betroffenen einwirken. Anschließend werden die Einflussfaktoren und deren Konsequenzen definiert (beispielsweise das Belohnen bei einer richtigen Beantwortung einer Quizfrage fördert die Motivation, die Interaktivität durch das Quiz das Engagement). Um die Einflussfaktoren zu nutzen, sodass die gewünschten Ziele erreicht werden, müssen bestimmte Voraussetzungen erfüllt sein (z. B. müssen die Videotools die Implementierung von interaktiven und Gamification-Elementen ermöglichen). Als Folge der vorgeschlagenen Entwurfsmuster wird eine bewährte Lösung angegeben, die den Ersteller*innen von Inhalten helfen soll, die Entwurfsmuster anzuwenden, um das gewünschte Ergebnis zu erzielen. In unserem Fall implementieren wir Quizzes oder Tests, die einen Belohnungsmechanismus zur Unterstützung von Lernergebnissen beinhalten. Die Erstellenden werden ermutigt, relevante Literatur und Best Practices zu berücksichtigen sowie ihre Zielgruppe zu befragen.

Basierend auf diesen Überlegungen soll im nächsten Schritt konkrete Entwurfsmuster im Rahmen einer Wirtschaftsinformatik-Grundlagenveranstaltung entwickelt werden, welche die Entwicklung von IGLV in der Praxis unterstützen können.

## Anwendung von Entwurfsmustern am Beispiel einer Wirtschaftsinformatik – Grundlagenveranstaltung

Um die Anwendung der Entwurfsmuster zu verdeutlichen und ihren Nutzen für die Entwicklung von IGLV evaluieren zu können, erfolgt die Entwurfsmusterentwicklung im Rahmen einer Grundlagenveranstaltung für Wirtschaftsinformatik. In der Lehrveranstaltung, welche als Flipped Classroom konzipiert ist, werden Lernvideos bereits zur Unterstützung im Lernprozess eingesetzt (Lehmann et al. 2015; Janson et al. 2017). Diese Lernvideos werden insbesondere im Rahmen einer Selbstlernphase zurückgegriffen, in welcher sich die Studierenden Lerninhalte selbstständig aneignen. Diese Lernvideos sollen im Zuge einer Überarbeitung des Lehr-Lernkonzepts der Veranstaltung überarbeitet und aktualisiert werden. Bei dieser Überarbeitung sollen die Entwurfsmuster als Grundlage für die Überarbeitung der Videoinhalte dienen. Das genaue Vorgehen wird in Tab. 4 dargestellt.

**Tab. 4:** Ablauf der IGLV Entwicklung in der Grundlagenveranstaltung

*1. Konzeptionsphase*	
Ziel	Erarbeitung von Struktur und Ablauf der Interaktionen
Inhalt	1) Brainstorming und Priorisierung von Ideen
	2) Planung der Interaktionen und Identifizierung von Haupt- und Nebeninformationen
	3) Entwicklung eines Flowcharts zur Ablaufplanung der Interaktionen
	4) Entwicklung eines Storyboards zur Integration der Interaktionen in den Videoablauf
Ergebnis	Ablaufplan des Lernvideos mit zugehörigen Interaktionen
*2. Durchführungsphase*	
Ziel	Entwicklung der Lernvideos
Inhalt	Lehrende führen Videoaufnahme durch. Hier ist auf die fünf Eckpunkte der Videoproduktion zu achten: 1) Bereitstellung des notwendige Equipments 2) Gute Ausleuchtung, 3) Positionierung der Kamera, 4) Nutzung des Storyboards, 5) Zusätzliche Bildschirmaufnahmen
Ergebnis	Videorohmaterial
*3. Nachbearbeitungsphase*	
Ziel	Integration von interaktiven und gamifizierten Elementen in das Lernvideo
Inhalt	Mittels geeigneter Software (siehe Tab. 2) werden die in der Konzeptionsphase entwickelten Interaktionen in das Lernvideo eingebaut. Überprüfung der Systematik der Interaktionen, falls vom Storyboard abgewichen worden ist
Ergebnis	Produziertes IGLV

Die Entwurfsmuster werden dabei in den drei Phasen des Erstellungsprozesses von Lernvideos eingesetzt: 1) Konzeptionsphase, 2) Durchführungsphase und 3) Nachbearbeitungsphase, wobei wir uns an dieser Stelle auf die Konzeptionsphase konzentrieren, da diese entscheidend für die systematische Integration der Interaktionselemente in die Lernvideos ist, oftmals vernachlässigt wird und dadurch zu den beschriebenen Problemen bei IGLV führt. Insgesamt konnten sieben Pattern entwickelt werden, wobei diese momentan noch weiter verfeinert und direkter auf bestimmte Interaktionen hin angepasst werden (siehe Zusatzmaterial online).

**Abb. 4:**
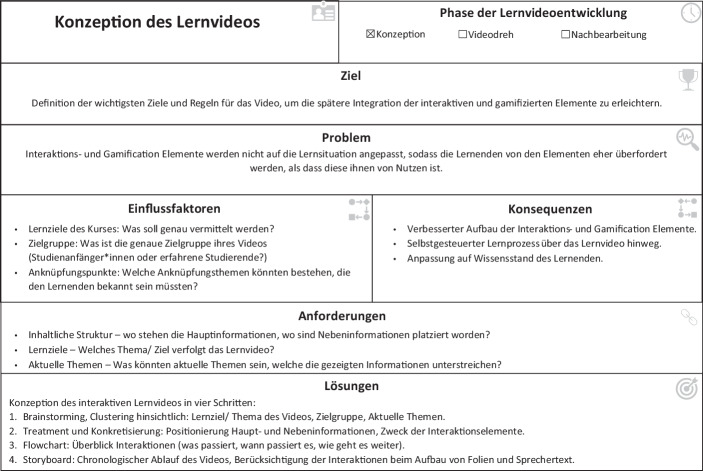
Entwurfsmuster für die Konzeptionsphase der IGLV

**Abb. 5:**
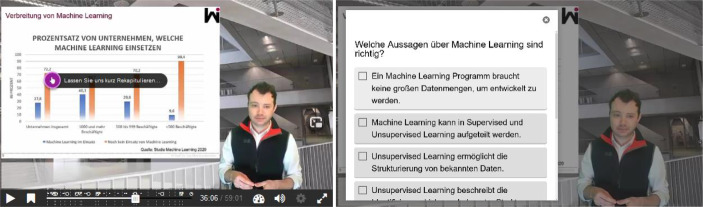
Beispiel eines verpflichtenden Wissenstest im Rahmen der Veranstaltung

Die Konzeptionsphase dient der Planung und Vorbereitung der IGLV. Die wichtigsten Schritte der Konzeptionsphase der IGLV werden im Entwurfsmuster „Konzeption des Lernvideos“ festgehalten (siehe Abb. 4). Die Entwurfsmuster beziehen sich dabei ausschließlich auf die Gestaltung der Interaktions- und Gamification Elemente im Video, nicht auf die generelle Gestaltung von Lernvideos, welche in unterschiedlichen Studien, beispielsweise von Lagerstrom et al. (2015), thematisiert worden sind. In der Konzeptionsphase werden die Grundlagen für die spätere Integration der Interaktionselemente geschaffen, welche in vier Unterpunkte gegliedert werden können. Zu Beginn der Konzeptionsphase werden mittels Brainstorming und Clusteringelementen möglichst viele Ideen gesammelt (1). Hierzu können beispielsweise Ideenworkshops oder auch ein Videocanvas (Ebner et al. 2021) genutzt werden. Ziel dieses Brainstormings ist die Entwicklung eines Grundverständnisses für die Zielgruppe des Lernvideos (beispielsweise welches Vorwissen vorhanden sein könnte oder welche thematischen Anknüpfungspunkte bestehen), der Identifizierung von relevanten Themen/Beispielen für die Ergänzung des Lerninhalts sowie der Entwicklung von Ideen für Interaktionen. Im nächsten Schritt (2) werden Haupt- und Nebeninformationen identifiziert, welche im Rahmen des Lernvideos adressiert werden sollen (vgl. Anf. 1). Da diese Informationen wichtig für das Erreichen des Lernziels des Videos sind, sollten an dieser Stelle Interaktionen zur Verdeutlichung und zu Wissenskontrolle eingesetzt werden (vgl. Anf. 2 und 3). Verpflichtende Wissenstest eignen sich dabei insbesondere an Stellen, an welchen Hauptinformationen vermittelt werden. Ein Beispiel für einen solchen verpflichtenden Wissenstest ist in Abb. 5 zu sehen. Hierdurch kann sichergestellt werden, dass Lernende die gezeigten Informationen auch verstanden und verinnerlicht haben. Bei Nebeninformationen können durch freiwillige Wissenstests das Verständnis für den Hintergrund der Informationen auf selbstgesteuerter Basis verbessert werden. Nachdem die Platzierung der Haupt- und Nebeninformationen durchgeführt worden ist, sollte über die Verknüpfung der Informationen mittels Interaktionselementen (3) nachgedacht werden. Beispielsweise ermöglicht die richtige Beantwortung eines Wissenstest das Überspringen bestimmter Teile des Lernvideos. Wird also der Wissenstest bei einer Hauptinformation richtig beantwortet, besteht ggf. keine Notwendigkeit die Nebeninformationen im Lernvideo anzuschauen. Zur Planung der Verknüpfungen bieten sich Flowcharts an. Diese Flowcharts dienen dabei der Übersichtlichkeit, an welchen Stellen welche Interaktionen platziert werden, was sie bewirken und wohin diese im Video führen sollen. Abschließend konzipieren wir ein Storyboard, welche alle gesammelten Informationen zusammenführt – den chronologischen Ablauf des Lernvideos, die Grobskizzierung des Sprechertextes, sowie die hierauf abgestimmten Interaktionselemente.

Die auf Grundlage der Entwurfsmuster entwickelten IGLV wurden dabei im Rahmen der Grundlagenveranstaltung evaluiert, um die Nützlichkeit und Gebrauchstauglichkeit der Muster belegen zu können. Die Ergebnisse dieser Evaluation wird im nächsten Kapitel dargestellt.

## Diskussion der Ergebnisse

Während im vierten Kapitel die konkrete Implementierung unser Entwurfsmuster für IGLV in einer Grundlagenveranstaltung zur Wirtschaftsinformatik dargestellt wurde, wird nun die Eignung des Ansatzes kritisch diskutiert und reflektiert, um hieraus Implikationen für Forschung und Praxis abzuleiten. Im Rahmen einer individuellen Lernphase, welche Teil des Flipped Classroom Konzepts ist, wurden interaktive und gamifizierte Lernvideos auf Basis der entwickelten Entwurfsmuster produziert. Vor und nach dieser individuellen Lernphase wurde eine Online-Umfrage mit 87 Studierenden durchgeführt, um qualitative Daten hinsichtlich der Eignung der Videos für das Konzept zu evaluieren und die Qualität der IGLV aus Sicht der Studierenden bewerten zu können. Hierzu wurden in einer Lerneinheit die bestehenden Lernvideos mittels der entwickelten didaktischen Entwurfsmuster mithilfe der Software h5p überarbeitet und weiterentwickelt. Die Wahl auf h5p erfolgte, weil die Software eine gute Integrationsmöglichkeit in das bestehende Lernmanagementsystem moodle bietet. Die interaktiven Lernvideos wurden von zwei erfahrenen Dozierenden entwickelt und anschließend von einem Tutor auf Verständlichkeit und Nachvollziehbarkeit überprüft.

Vor dem Beginn der Lerneinheit wurden die bisherigen Erfahrungen mit den bestehenden, nicht interaktiv aufbereiteten Videos erhoben. Während und nach der Lerneinheit, welche insgesamt aus fünf Videos mit einer gesamten Laufzeit von ca. 60 min bestand, konnten die Studierenden direktes Feedback zur Gestaltung und zum Aufbau der Interaktionselemente und der Videos an sich geben. Gleichzeitig sollten die Studierenden ihre eigene Meinung zu den IGLV im Vergleich zu den bekannten nicht interaktiv gestalteten Lernvideos bewerten sowie den zusätzlichen Aufwand zur Bearbeitung der interaktiven und gamifizierten Elemente beurteilen. Die Daten der Studierenden wurden anonym erhoben. Sie werden im Folgenden anhand der skizierten Potenziale und Limitierungen von IGLV dargestellt.

Kernfunktion der entwickelten Entwurfsmuster ist die Darstellung von Gestaltungswissen für die Entwicklung von didaktisch hochwertigen interaktiven und gamifizierten Lernvideos mit dem Ziel, die Interaktion innerhalb der Lernvideos über alle Interaktionsformen hinweg zu steigern, um dadurch den Lernerfolg der Studierenden zu verbessern. Unter Bezugnahme auf die Theorie der kognitiven Last sollten die Interaktionen dabei so angelegt werden, dass diese den Lernprozess der Studierenden sinnvoll unterstützen und diese durch die Interaktionen nicht unnötig abgelenkt werden (Koć-Januchta et al. 2020). Während der Evaluation konnte gezeigt werden, dass die auf Basis der Entwurfsmuster entwickelten Interaktionselemente einen sinnvollen Beitrag zur Unterstützung der Studierenden leisten können. Die folgenden Auszüge aus der studentischen Evaluation geben deutliche Hinweise darauf, dass die Interaktions- und Spiel-Elemente die gewünschten Effekte erzielt haben:

In den Lernvideos haben mir die Interaktionen in den Quizfragen sehr gut gefallen, da man durch die stetig gestellten Fragen aufmerksamer war und diese als Gedächtnisstütze dienten.

Durch die Elemente innerhalb der Lernvideos konnte die Interaktion gefördert werden, was dazu geführt hat, dass die Studierenden dem Lehrenden länger aufmerksam zugehört haben und auch zufriedener im Vergleich zu den nicht interaktiv gestalteten Videos waren:

Die Interaktivität war der springende Grund, warum ich den Exkurs besser fand als die vorherigen Videos.

Gleichzeitig zeigen Studien aber auch, dass in vielen Fällen die Interaktionen nicht systematisch aufgebaut wurden (Koć-Januchta et al. 2020) und Lehrende vorzugsweise zu viele Interaktionen einbauen als zu wenige. Dies kann den Aufwand für die Betrachtung der Lernvideos erhöhen, was zu Problemen für das Verständnis des Inhalts führen kann:

Das Lösen der Aufgaben war deutlich anspruchsvoller als die vorherigen Lernzyklen.

Diesen Umstand müssen die Entwurfsmuster noch stärker berücksichtigen und eine klarere Erläuterung bereitstellen, wie unterschiedliche Interaktionselemente zusammenwirken und welche Interaktionselemente bei welchen Lernanlässen eingesetzt werden sollten. Daher kann es zielführend sein, bei der Auswahl der Interaktionselemente eine Reihe unterschiedlicher Elemente zu nutzen, welche sich vom Aufwand für den Lernenden her unterscheiden. Beispielsweise ist der Aufwand der Beantwortung eines Multiple Choice Tests im Vergleich mit einer Textaufgabe relativ gering. Gleichzeitig könnte der gefühlte Aufwand für die Beantwortung der Fragen durch einen noch gezielteren Einsatz von Spiel-Elementen verringert werden. Durch die Nutzung der Software h5p im Zusammenspiel mit der Lernsoftware Moodle war der Einsatz von Spiel-Elementen z. T. seitens des Programmes eingeschränkt. Wir vermuten, dass sich ein noch gezielterer Einsatz dieser Elemente positiv auf die Nutzung der Interaktionselemente auswirken könnte, was sich bereits auch in ähnlichen Studien gezeigt hat (Deng et al. 2020). Die Erfahrung hat gezeigt, dass die Entwicklung von IGLV sehr aufwendig ist. Insbesondere die Konzeptionsphase kann sehr viel Zeit in Anspruch nehmen, um hochwertige Wissenstests und Verknüpfungen innerhalb der Lernvideos (und auch lernvideoübergreifend) zu entwickeln. Dieser Aufwand rentiert sich jedoch langfristig, da die Videos nicht für jedes Semester neu gedreht werden müssen. Gleichzeitig kann durch die Nutzung der Interaktionselemente die Halbwertszeit der Videos erhöht werden, da diese auch nachträglich eingebaut und aktualisiert werden können, ohne bereits gedrehte Lernvideo neu aufnehmen zu müssen.

Gleichzeitig hat das Konzept von IGLV unter Berücksichtigung gewisse Limitierungen. Ein wichtiger Punkt ist die notwendige IT-Unterstützung bei der Gestaltung der Videos. Diese ermöglicht erst die Integration der interaktiven und gamifizierten Elemente zur Unterstützung der Lernenden. Vor diesem Hintergrund sind die Integrationsmöglichkeiten stark abhängig vom gewählten Anbietenden und von der gewählten Lernplattform (vgl. mit den angebotenen Interaktionselementen in Tab. 2), was zu Einschränkungen bei der Implementierung und beim Aufbau der Interaktionen führen kann. Generell sind diese Interaktionsmöglichkeiten oftmals von der jeweiligen Institution vorgegeben, weil sie mit dem vorhandenen Lernmanagementsystem kompatibel sein muss. Es erwies sich in unserem Fall als sehr wichtig, sich bereits in der Konzeptionsphase vertiefend mit den Möglichkeiten der gewählten Interaktionssoftware vertraut zu machen. Insbesondere ist an dieser Stelle festzustellen, dass die Entwicklung der Interaktionselemente eine gewisse Erfahrung benötigt, um ein gut zusammenhängendes Konzept für den Aufbau der Interaktionen entwickeln zu können. Darüber hinaus stellen Interaktionselemente einen zusätzlichen Aufwand bei der Betrachtung der Lernvideos für die Studierenden dar, welcher bei der Konzeption der Videos berücksichtigt werden sollte.

Für mich war die Bearbeitung der Aufgaben sehr Zeitaufwendig.

Zusammenfassend konnte gezeigt werden, dass insbesondere durch die Covid-19-Krise aufkommende intensive Nutzung von Lernvideos in der Hochschullehre die Integration von interaktiven und spielerischen Elementen einen Beitrag dazu leisten kann, die Interaktionen von Studierenden in allen Bereichen zu fördern:

Die Interaktion, wie zum Beispiel die Quizfragen etc. haben die Aufmerksamkeit während der Lernphase verstärkt und war eine schöne Abwechslung zu dem, was wir im Moment ständig machen, nämlich stupide nur Videos anzuschauen und Mitschriften zumachen.

## Zusammenfassung und Ausblick

Der vorliegende Beitrag beschreibt die problemorientierte Gestaltung von Entwurfsmustern für die IGLV und führt auf, wie die Qualität dieser Lernvideos systematisch gesteigert werden kann. Die Ergebnisse des Beitrags verdeutlichen, wie interaktive und gamifizierte Elemente die Interaktion bei der Nutzung von Lernvideos verbessern und dadurch Lernende in ihrem Lernprozess unterstützt werden können. Die Ergebnisse sind dabei für Wissenschaftler*innen, Dozierenden und andere Praktizierenden gleichermaßen von praktischer Relevanz, da sie Erkenntnisse dazu liefern, mit welchen Mitteln die Interaktion innerhalb von Lernvideos gesteigert werden kann. Gleichzeitig kann eine Einschätzung vermittelt werden, wie diese Interaktionen bestmöglich in den Videos platziert werden sollten. Ausgehend von der Interaktionstheorie, dem Gamification Konzept sowie der Theorie der kognitiven Last wurden zum einen theoretische Anforderungen erhoben, welche auch durch praktische Anforderungen aus Fokusgruppenworkshops und Umfragen ergänzt worden sind. Diese Anforderungen wurden anschließend in Entwurfsmuster übersetzt, um Lehrende bei der Entwicklung ihrer eignen IGLV zu unterstützen. Anschließend wurde die Entwicklung und der Einsatz dieser Entwurfsmuster am Beispiel einer Grundlagenveranstaltung für Wirtschaftsinformatik verdeutlicht. Dabei wurde aufgezeigt, welchen Nutzen der Einsatz von IGLV in der Lehre im Vergleich zu herkömmlichen Lernvideos besitzen kann. Ausgehend von unseren Erkenntnissen ist die Nutzung von Entwurfsmustern zur Entwicklung von IGLV ein gebrauchstaugliches Mittel zur Unterstützung von Lehrenden bei der Entwicklung qualitativ hochwertiger interaktiver und gamifizierter Lernvideos. Zukünftiger Forschungsbedarf besteht dabei insbesondere in der kontinuierlichen Weiterentwicklung der Entwurfsmuster in dem Sinne, dass diese besser in das jeweilige Lehr-Lernszenario eingebunden werden können. Insbesondere Interaktionsmöglichkeiten, welche über ein einzelnes Lernvideo hinausgehen und mehrere Lernvideos zu einem großen Lehr-Lernszenario verbinden, konnten im Rahmen unseres Beitrags nicht ausreichend betrachtet werden. Darüber hinaus wurden in der hier dargestellten Iteration der Entwurfsmuster nur qualitative Daten vorgestellt. Um eine weitere Validierung zu ermöglichen, und um genauere Ergebnisse hinsichtlich der Förderung des Lernerfolgs, der Motivation und des Engagements aufzeigen zu können, planen wir im Rahmen der nächsten Iteration eine quantitative Erhebung mittels A/B Experiment. Darüber hinaus konnte festgestellt werden, dass es noch mehr Forschungsbedarf hinsichtlich der Gestaltung der einzelnen Interaktionselemente gibt und welche Wirkung die einzelnen Elemente auf die Interaktion besitzen. Beispielsweise ist denkbar, dass sich bestimmte Interaktionselemente besonders für die Verbesserung einer spezifischen Interaktion eignen.

## Supplementary Information

Finale Pattern für die Konstruktionsphase von interaktiven und gamifizierten Lernvideos
