# Supplementary figures and images for: Supporting Digital Education Processes Through Interactive Gamified Learning Videos—How Innovative Learning Videos can Increase Motivation and Learning Success
Source: HMD Prax Wirtsch Inform. 2021 Oct 11;58(6):1483–503. [Article in German] doi: 10.1365/s40702-021-00798-w (PMC8504096; doi:10.1365/s40702-021-00798-w)

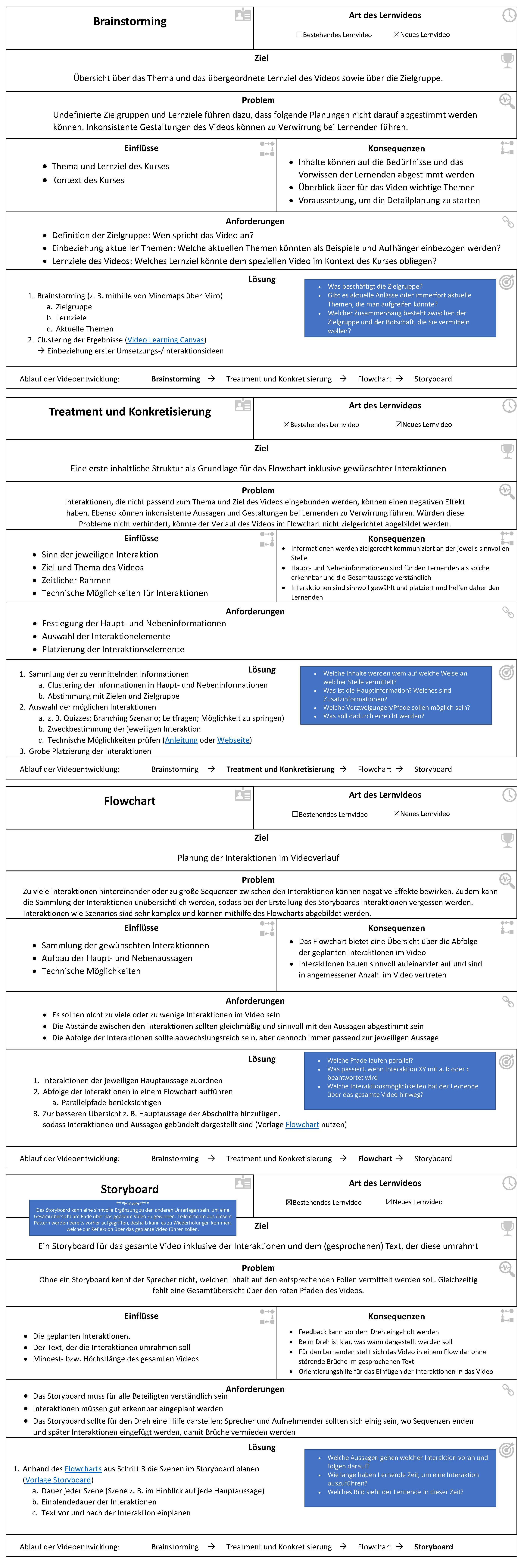

Supplement: Supplementary file 1 — Finale Pattern für die Konstruktionsphase von interaktiven und gamifizierten Lernvideos [file 40702_2021_798_MOESM1_ESM.jpg]
